# Nutrition Education in Greek Secondary School Textbooks: A Content Analysis of Coverage and Thematic Orientation

**DOI:** 10.3390/nu18081257

**Published:** 2026-04-16

**Authors:** Antonios Emmanouil Chronakis, Emilia Vassilopoulou, Elisabeth Vardaka, Athanasios Papadopoulos

**Affiliations:** 1 Department of Nutritional Sciences and Dietetics, School of Health Sciences, International Hellenic University, 57400 Thessaloniki, Greece; antonishronakis@yahoo.com (A.E.C.); evardaka@ihu.gr (E.V.); 2 Pediatric Area, Fondazione IRCCS Ca’ Granda-Ospedale Maggiore Policlinico, 20122 Milan, Italy; 3 Department of Clinical Sciences and Community Health, Università Degli Studi di Milano, 20122 Milan, Italy; 4 Department of Life Sciences, School of Life and Health Sciences, Universiy of Nicosia, 24005 Nicosia, Cyprus

**Keywords:** nutrition education, secondary school textbooks, content analysis, food literacy, health education, curriculum evaluation

## Abstract

**Background**: In Greece, nutrition education is not taught as an independent subject but is incorporated across multiple disciplines. Objective: To evaluate the extent, distribution, and thematic orientation of nutrition-related content in Greek secondary school textbooks. **Methods**: A systematic content analysis was conducted on all officially approved textbooks used in Greek secondary education during the academic year 2022–2023. A total of 164 textbooks (26,914 pages) were analyzed. Nutrition-related references were systematically identified using predefined keywords and categorized into thematic domains. Coding was performed independently by two researchers (Cohen’s κ = 0.84). **Results**: A total of 1426 nutrition-related references were identified. Nutrition-related content was unevenly distributed, with 57.1% in Gymnasium textbooks and 42.9% in Lyceum textbooks. When normalized, Gymnasium textbooks contained 7.47 references per 100 pages compared with 3.82 in Lyceum. Content was primarily focused on biological concepts (27.1%) and health/disease prevention (20.3%), while behavioral (16.5%), psychosocial (16.2%), and sustainability-related (19.8%) dimensions were less represented. No references addressed skills-based nutrition education. **Conclusions**: Nutrition education in Greek textbooks is present but fragmented and predominantly biologically oriented. The lack of behavioral and skills-based content suggests the need for more comprehensive and interdisciplinary educational approaches.

## 1. Introduction

Nutrition education is widely recognized as a critical public health strategy for the promotion of healthy eating behaviors and the prevention of non-communicable disease [1]. Dietary patterns established during childhood and adolescence tend to persist into adulthood, influencing long-term health outcomes, including obesity, cardiovascular disease, diabetes, and metabolic disorders [2,3]. Schools constitute a key environment for the development of nutrition-related knowledge, attitudes, and behaviors, as they provide structured learning experiences during formative years and reach nearly the entire youth population [4].

Despite the growing international emphasis on school-based nutrition education, many educational systems continue to address nutrition in a fragmented and predominantly biomedical manner, often limiting instruction to isolated biological concepts such as nutrients, digestion, or energy balance [5]. Contemporary frameworks of nutrition education, however, advocate for broader, interdisciplinary approaches that integrate behavioral, social, environmental, and cultural dimensions of food and eating [6,7]. These approaches emphasize food literacy, critical thinking, sustainable diets, and the development of lifelong healthy eating behaviors rather than the mere transmission of factual knowledge.

In Greece, nutrition-related content is not offered as an autonomous subject within secondary education. Instead, nutrition-related content is dispersed across multiple disciplines, including biology, home economics, physical education, and health education [8]. While this cross-curricular structure has the potential to support interdisciplinary learning, it also risks producing inconsistencies, content gaps, and conceptual fragmentation. The extent to which Greek secondary school textbooks provide coherent, comprehensive, and pedagogically adequate nutrition education has not been systematically examined [9].

Textbooks remain the dominant instructional resources in Greek public education and play a central role in shaping both teaching practices and student learning experiences. They do not merely convey scientific information but also reflect curricular priorities, educational philosophies, and implicit assumptions regarding health, food, and lifestyle [10]. Consequently, the analysis of textbook content offers valuable insight into the educational messages students receive about nutrition and health.

International research has demonstrated that school textbooks often emphasize biological or medicalized aspects of nutrition while underrepresenting behavioral, psychosocial, and socio-environmental determinants of dietary practices [11]. Furthermore, limited attention is frequently given to contemporary nutrition challenges, such as childhood obesity, food sustainability, media influences, and the development of critical food-related decision-making skills [12]. These shortcomings may undermine the effectiveness of school-based educational material and restrict its potential impact on students’ everyday behaviors.

To date, no comprehensive content analysis has examined how nutrition education is presented across Greek secondary school textbooks. Addressing this gap is essential for evaluating whether current educational materials align with modern principles of nutrition education and public health promotion. In addition, understanding how nutrition is framed within school curricula is increasingly recognized as an important determinant of food literacy and long-term dietary behavior among adolescents, which may ultimately influence population-level dietary patterns and chronic disease prevention.

Therefore, the aim of this study was to systematically analyze nutrition-related content in Greek secondary school textbooks to assess its extent, distribution, and thematic orientation. This study is informed by contemporary nutrition education frameworks, including food literacy and behavior-oriented education models, which emphasize not only biological knowledge but also behavioral, psychosocial, and environmental determinants of dietary practices [13,14]. Furthermore, European policy initiatives such as the EU School Scheme [15] and the Farm to Fork Strategy [16] highlight the importance of integrated nutrition education within school systems [16]. These frameworks provide the conceptual basis for evaluating textbook content.

## 2. Methods

### 2.1. Study Design

This study employed a mixed-methods content analysis design, integrating quantitative frequency analysis with qualitative thematic interpretation. Mixed-methods content analysis has been widely used in nutrition education research to evaluate curricular materials, educational messages, and the alignment of teaching resources with contemporary public health nutrition frameworks. The methodological approach was selected to systematically examine the extent, distribution, and pedagogical orientation of nutrition-related content in Greek secondary school textbooks.

Content analysis was chosen as it allows for both objective quantification of manifest content and in-depth interpretation of latent educational meanings, making it particularly suitable for evaluating curricular materials and instructional resources.

### 2.2. Sample of Textbooks

The study included all officially approved textbooks used in Greek secondary education during the academic year 2022–2023. In total, 164 textbooks were analyzed (84 Gymnasium, 80 Lyceum), corresponding to 26,914 pages (Supplementary Table S1).

Textbooks were retrieved from the national educational publishing system and included all compulsory subjects in which nutrition-related references were identified.

The final sample comprised textbooks from (a) Gymnasium (Grades 7–9); (b) Lyceum (Grades 10–12).

Multiple subject areas were represented, including biology, health-related courses, and interdisciplinary subjects where nutrition content was embedded. All volumes officially distributed to students during the study period were eligible for inclusion.

### 2.3. Identification of Content and Reliability

Nutrition-related content was identified using predefined keywords (e.g., nutrition, diet, food, nutrients) and contextual interpretation. Ambiguous cases were discussed between coders and classified through consensus. Coding was performed independently by two researchers. A pilot coding phase (10% of dataset) was conducted. Inter-rater agreement was high (Cohen’s κ = 0.84). Discrepancies were resolved through discussion.

### 2.4. Unit of Analysis

The unit of analysis was defined as any discrete reference to nutrition, food, diet, or health-related dietary issues. Units included paragraphs, thematic sections, activities, tables, figures, and supplementary text that explicitly or implicitly addressed nutrition-related topics.

Each unit was coded once, even when multiple nutrition concepts were present, to avoid duplication of frequency counts while allowing qualitative co-coding within thematic categories.

### 2.5. Coding Framework and Thematic Categories

A coding framework was developed deductively, informed by contemporary nutrition education literature and the conceptual structure of the doctoral research. Categories were designed to capture both scientific content and educational orientation.

The main thematic categories included: (1) Nutritional science and biological concepts (e.g., nutrients, digestion, metabolism, energy balance); (2) Health and disease prevention (e.g., obesity, chronic disease, dietary guidelines); (3) Dietary behavior and lifestyle (e.g., eating habits, food choices, physical activity); (4) Psychosocial and cultural dimensions of food (e.g., social influences, body image, media); (5) Food systems and sustainability (e.g., food production, environmental impact, food quality); (6) Educational and skills-based content (e.g., critical thinking, label reading, meal planning).

Skills-based nutrition education was defined as content promoting practical competencies (e.g., meal planning, food preparation, label reading, decision-making).

Additionally, each unit was classified according to: (a) Educational level (Gymnasium/Lyceum); (b) Subject area; (c) Type of presentation (theoretical text, activity-based, visual material).

### 2.6. Data Collection Procedure

All textbooks were systematically reviewed page by page. Nutrition-related content was first identified and recorded in a structured coding sheet. Each identified unit was catalogued by textbook title, grade level, subject, and page number.

Following initial screening, all units were coded into the predefined thematic categories. Coding was performed manually to allow close contextual interpretation of educational intent, conceptual depth, and interdisciplinary orientation.

### 2.7. Quantitative Analysis

Quantitative analysis focused on descriptive frequency measures. The number of nutrition-related references was calculated: (a) per textbook; (b) per grade level; (c) per thematic category; (d) per educational level (Gymnasium vs. Lyceum).

Relative distributions and proportional representations were used to identify dominant themes, underrepresented areas, and imbalances across the curriculum.

Results were summarized in frequency tables and comparative graphs to illustrate the distribution patterns of nutrition education content.

### 2.8. Qualitative Analysis

Qualitative thematic analysis was conducted to examine: (a) the conceptual framing of nutrition; (b) the pedagogical orientation (informational vs. skill-based); (c) the degree of interdisciplinarity; (d) the alignment with contemporary nutrition education principles.

Special attention was given to identifying whether content promoted holistic nutrition education (behavioral, social, preventive) or remained confined to biomedical descriptions.

### 2.9. Rigor and Trustworthiness

To enhance methodological rigor, the coding framework was established prior to analysis and applied consistently across all textbooks. Categories were clearly defined to minimize ambiguity. Repeated reviews of coded material were conducted to ensure internal consistency and confirm thematic coherence. Any ambiguous cases were re-examined within their educational context before final categorization.

### 2.10. Ethical Considerations

The study analyzed publicly available educational materials and did not involve human participants. Therefore, formal ethical approval was not required.

## 3. Results

### 3.1. Overall Distribution of Nutrition-Related Content

Across the analyzed textbooks, a substantial number of references to nutrition-related issues were identified. These references varied considerably in frequency and depth across school levels and subject areas.

The total number of textbooks analyzed, total pages reviewed, and total nutrition-related references identified are summarized in Table 1.

Overall, nutrition content appeared unevenly distributed, with a higher concentration in specific subjects and grade levels, while several textbooks contained minimal or no references to nutrition-related topics.

### 3.2. Distribution by Educational Level (Gymnasium vs. Lyceum)

A comparative analysis between Gymnasium and Lyceum textbooks revealed notable differences in both the quantity and thematic focus of nutrition-related content.

As presented in Table 2, Gymnasium textbooks accounted for a larger proportion of total references, primarily concentrated in introductory biological and health-related contexts. In contrast, Lyceum textbooks showed fewer overall references, which were more fragmented and predominantly embedded within broader scientific discussions rather than explicitly framed as nutrition education.

When normalized, Gymnasium textbooks contained 7.47 references per 100 pages, compared with 3.82 in Lyceum textbooks (Table 3).

### 3.3. Thematic Categorization of Nutrition Content

All identified references were classified into thematic categories reflecting the conceptual orientation of nutrition education.

The distribution of references by thematic category is presented in Table 4.

The majority of references addressed biological and nutritional science concepts (e.g., nutrients, digestion, metabolism), followed by content related to health and disease prevention. Substantially fewer references addressed dietary behavior, psychosocial dimensions of food, or food systems and sustainability. No references were classified as skills-based nutrition education according to the predefined coding criteria.

Educationally oriented themes, such as the development of food-related skills, critical thinking, and informed dietary decision-making, were infrequently represented.

### 3.4. Grade-Level Patterns

When examined by grade level, distinct patterns emerged. As shown in Table 5, nutrition-related references were more frequent in lower secondary grades, with a gradual decline observed in upper secondary education.

In Gymnasium textbooks, references were mainly introductory and informational, focusing on basic biological knowledge. In Lyceum textbooks, nutrition content was more sporadic and often disconnected from everyday dietary practices.

### 3.5. Qualitative Thematic Analysis

Qualitative analysis indicated that nutrition education within the textbooks was predominantly framed through a biomedical lens, emphasizing physiological processes and disease prevention. Holistic perspectives, integrating behavioral, social, cultural, and environmental determinants of nutrition, were limited. Several textbooks contained minimal or no nutrition references, while others concentrated content within isolated sections, supporting the observed uneven distribution.

Explicit connections between nutrition knowledge and students’ daily food choices were rare. Furthermore, interdisciplinary linkages across subjects were minimal, and few activities promoted critical engagement, skills development, or reflective learning.

## 4. Discussion

The present study provides the first systematic analysis of nutrition-related content in Greek secondary school textbooks, revealing substantial imbalances in both thematic coverage and pedagogical orientation. Textbooks in Greece are developed by expert committees under the supervision of the Ministry of Education and are periodically revised. Their use is mandatory in public education. Although nutrition topics were present across multiple subjects and grade levels, their distribution was uneven and their educational framing predominantly biomedical. This pattern suggests that nutrition education within Greek secondary education is largely confined to scientific description rather than positioned as a comprehensive educational process aimed at shaping dietary behaviors and food-related competencies.

A central finding of this study was the predominance of biological and physiological content, including nutrients, digestion, metabolism, and disease prevention. While such knowledge constitutes an essential foundation of nutrition education, its dominance to the exclusion of broader dimensions limits the educational potential of school-based nutrition instruction [4,17]. The observed emphasis on biological content contrasts with European policy priorities such as the Farm to Fork Strategy, which promotes sustainable diets and food literacy [16]. This discrepancy suggests potential misalignment between educational materials and broader public health strategies.

The absence of skills-based content is particularly notable given its central role in contemporary nutrition education frameworks, where practical competencies are considered essential for translating knowledge into behavior. Contemporary nutrition education frameworks emphasize the integration of behavioral, social, and environmental determinants of eating, along with the development of critical food literacy skills. The limited representation of dietary behavior, psychosocial influences, and sustainability observed in the analyzed textbooks indicates a narrow conceptualization of nutrition, which may be insufficient to fully support meaningful behavior change among adolescents [18].

The results further demonstrated that explicit connections between nutrition knowledge and students’ everyday dietary practices were scarce. Few textbook units addressed practical skills such as informed food choice, label interpretation, meal planning, or critical evaluation of food-related media messages. This absence of skills-based content weakens the capacity of textbooks to function as tools for health promotion. Previous research in school-based nutrition education highlights that information-only approaches are rarely effective unless accompanied by experiential, reflective, and behaviorally oriented learning opportunities [19,20]. The findings of the present study suggest that Greek secondary school textbooks largely appear to have limited incorporation of these principles. Similar patterns have been reported internationally, where nutrition education remains predominantly knowledge-based rather than behavior-oriented, highlighting a persistent gap between educational content and public health priorities [21,22,23].

Another important finding concerns the limited interdisciplinary integration of nutrition topics. Although nutrition-related references appeared in several subjects, they were rarely coordinated across disciplines or framed within a unified educational narrative. This fragmentation may hinder students’ ability to conceptualize nutrition as a multifaceted issue encompassing health, culture, environment, and personal responsibility [24]. International literature increasingly supports interdisciplinary curriculum models that connect biological knowledge with social sciences, environmental education, and health promotion [25]. The lack of such integration within Greek textbooks highlights a missed opportunity to foster holistic understanding and systems-based thinking around food and nutrition.

Differences between educational levels further reinforce these concerns. Nutrition-related references were more frequent in Gymnasium textbooks and decreased substantially in Lyceum. Moreover, upper secondary content was often embedded within advanced scientific contexts, reducing its relevance to students’ daily experiences. Adolescence represents a critical developmental period during which dietary autonomy increases and long-term habits are consolidated [26,27]. The reduced presence and fragmented nature of nutrition education in Lyceum textbooks may limit the potential role of school to support students at a stage when such guidance is particularly necessary [12,28].

Taken together, these findings indicate that Greek secondary school textbooks do not fully reflect contemporary paradigms of nutrition education that emphasize empowerment, food literacy, and lifelong health promotion. Instead, they largely reproduce a traditional biomedical model focused on factual transmission [29]. This orientation may reflect structural limitations in curricular representation; however, no conclusions can be drawn regarding intervention effectiveness of school-based nutrition initiatives in addressing complex public health challenges such as childhood obesity, unhealthy dietary patterns, and the influence of modern food environments [30]. Although these findings reflect structural characteristics of textbook content, no conclusions can be drawn regarding educational effectiveness or student outcomes. Similar findings of biologically oriented nutrition education have been reported in other educational systems, where limited emphasis is placed on behavioral and skills-based components [31].

The implications of this study are primarily curricular and pedagogical. Curriculum developers and textbook authors should consider reorienting nutrition content toward integrative educational frameworks that combine scientific knowledge with behavioral, psychosocial, and sustainability perspectives. Embedding nutrition education across subjects through coherent learning objectives, authentic activities, and critical engagement tasks may enhance both relevance and impact [32]. Furthermore, aligning textbook content with contemporary international guidelines on nutrition education could strengthen the role of schools in promoting healthy and sustainable dietary behaviors [33].

Several limitations should be acknowledged. This study evaluates textbook content only and does not assess teaching practices or student behavior. Consequently, the findings reflect the intended curriculum rather than enacted practice. Future research should explore how teachers interpret and utilize textbook material and how students engage with nutrition-related content. Additionally, comparative studies across countries and educational systems could further contextualize the Greek case and support the development of evidence-based curriculum reforms.

Despite these limitations, the present study offers critical insight into the current status of nutrition education in Greek secondary schools. By systematically documenting thematic emphases and educational gaps, it provides an empirical foundation for curriculum evaluation and future educational planning. Strengthening nutrition education within textbooks represents an essential step toward equipping young people with the knowledge, skills, and critical capacities required to navigate contemporary food environments and to adopt healthier lifelong dietary behaviors.

From a public health perspective, strengthening nutrition education within school curricula may represent an important strategy for improving population dietary behaviors. Schools constitute one of the few environments capable of reaching nearly all adolescents and therefore play a key role in the development of food literacy and long-term health-related behaviors. Aligning textbook content with contemporary nutrition education frameworks could enhance the effectiveness of school-based interventions aimed at promoting healthier and more sustainable dietary practices.

## 5. Conclusions

This study provides the first systematic examination of nutrition-related content in Greek secondary school textbooks and reveals significant limitations in both thematic coverage and educational orientation. Although nutrition topics are present across subjects and grade levels, their uneven distribution and predominant biomedical framing may constrain the capacity of textbooks to function as effective tools for comprehensive nutrition education.

The findings indicate that current textbook content emphasizes biological concepts and disease-related information while underrepresenting behavioral, psychosocial, and sustainability dimensions of food and nutrition. The limited presence of skills-based and interdisciplinary content suggests that students may not be adequately supported in developing food literacy, critical thinking, and practical competencies essential for healthy dietary decision-making.

These results underscore the need for more comprehensive and interdisciplinary approaches to nutrition education. Integrating holistic, behavior-oriented, and interdisciplinary approaches into secondary school textbooks may strengthen the role of formal education in promoting lifelong healthy and sustainable eating behaviors among adolescents. Strengthening the role of school curricula in nutrition education may therefore contribute to broader public health strategies aimed at improving dietary behaviors among future generations.

## Figures and Tables

**Table 1:** Textbooks, pages reviewed, and total nutrition-related references.

Educational Level	Number of Textbooks	Pages Reviewed	Nutrition-Related References (*n*)
Gymnasium	84	10.903	814
Lyceum	80	16.011	612
Total	164	26.914	1426

**Table 2:** Distribution of nutrition-related references by educational level.

Educational Level	References (*n*)	Percentage (%)
Gymnasium	814	57.1
Lyceum	612	42.9
Total	1426	100.0

**Table 3:** Normalization of nutrition-related references per 100 pages.

Educational Level	References per 100 Pages
Gymnasium	7.47
Lyceum	3.82

**Table 4:** Distribution of nutrition-related references by thematic category.

Thematic Category	References (*n*)	Percentage (%)
Nutritional science/biological concepts	387	27.1
Health and disease prevention	290	20.3
Dietary behavior and lifestyle	235	16.5
Psychosocial and cultural dimensions	231	16.2
Food systems and sustainability	283	19.8
Skills-based/educational content	0	0.0
Total	1426	100

**Table 5:** Nutrition-related references by grade level.

Grade	Number of References (*n*)	Main Thematic Emphasis
A Gymnasium	408	Harmful habits/disease links; core healthy habits
B Gymnasium	202	Cultural-historical/celebratory food references
C Gymnasium	152	Food adequacy (under/over-supply) and related issues
A Lyceum	155	Food adequacy and quantity-related concepts
B Lyceum	237	Food groups; unhealthy patterns (e.g., fast food)
C Lyceum	175	Food adequacy with selected food-group emphasis

## Data Availability

Upon request from the corresponding authors.

## Supplementary Materials

The following supporting information can be downloaded at: https://www.mdpi.com/article/10.3390/nu18081257/s1, Table S1: Nutrition-Related Learning Objects from the “Photodentro” National Educational Repository. The table presents the 69 learning objects (LOs) related to nutrition identified in the Greek National Educational Content Aggregator “Photodentro”.
