# Supplementary material for: Nutrition Education in Greek Secondary School Textbooks: A Content Analysis of Coverage and Thematic Orientation
Source: Nutrients. 2026 Apr 16;18(8):1257. doi: 10.3390/nu18081257 (PMC13119464; doi:10.3390/nu18081257)
Supplement: Supplementary file 1 [file nutrients-18-01257-s001.zip › nutrients-4220652-supplementary.pdf]

nutrients

MDPI

Supplementary Table S1: Nutrition-Related Learning Objects from the "Photodentro" National Educational Repository. The table presents the 69 learning objects (LOs) related to nutrition identified in the Greek National Educational Content Aggregator "Photodentro".

ΚΩΔΙΚΟΣ ΑΡΙΘΜΟΣ	ΤΙΤΛΟΣ	ΔΙΕΥΘΥΝΣΗ ΚΑΡΤΕΛΑΣ	ΕΚΠΑΙΔΕΥΤΙΚΗ ΒΑΘΜΙΔΑ	ΤΥΠΟΣ Μ.Α	ΘΕΜΑΤΙΚΗ ΠΕΡΙΟΧΗ
**8522/23**	Υγιεινή διατροφή	http://photodentro.edu.gr/aggregator/lo/photodentro-educationalvideo-8522-23	Γυμνάσιο ΓΕΛ	Βίντεο	Αγωγή Υγείας
**8526/7910**	Ισορροπημένη διατροφή	http://photodentro.edu.gr/aggregator/lo/photodentro-aggregatedcontent-8526-7910	ΓΕΛ	Εκπαιδευτικό σενάριο - Σχέδιο μαθήματος	Φυσική αγωγή
**8521/7811**	Μεσογειακή Διατροφή	http://photodentro.edu.gr/aggregator/lo/photodentro-lor-8521-7811	Δημοτικό Γυμνάσιο	Ανοιχτή δραστηριότητα, Ιστοσελίδα	Νέα Ελληνική Γλώσσα, Θέματα, Αθλητισμός, Διατροφή
**8522/25**	Προσχολική ηλικία - Διατροφή	http://photodentro.edu.gr/aggregator/lo/photodentro-educationalvideo-8522-25	Γυμνάσιο ΓΕΛ	Βίντεο	Αγωγή Υγείας
**8521/6787**	Διατροφή - Τροφή, ενέργεια και διατροφική πυραμίδα	http://photodentro.edu.gr/aggregator/lo/photodentro-lor-8521-6787	Γυμνάσιο ΓΕΛ ΕΠΑ.Λ	Εκπαιδευτικό σενάριο - Σχέδιο μαθήματος	Χημεία, Βιοχημεία
**8521/6988**	Είμαστε ό,τι τρώμε	http://photodentro.edu.gr/aggregator/lo/photodentro-lor-8521-6988	Γυμνάσιο, ΓΕΛ, ΕΠΑ.Λ	Ανοιχτή δραστηριότητα, Διερεύνηση, Ιστοσελίδα	Νέα Ελληνική Γλώσσα, Αθλητισμός, Διατροφή, Νέα Ελληνική Γλώσσα, Κειμενικοί τύποι, Επιχειρηματολογία

**8522/811**	Μπατζίνα	http://photodentro.edu.gr/aggregator/lo/photodentro-educationalvideo-8522-811	Προσχολική<br>Δημοτικό Γυμνάσιο<br>ΕΠΑ.Λ	Βίντεο	Αγωγή Υγείας
**8526/7189**	Ελαιώνας στη διαδρομή προς Πελοπόννησο	http://photodentro.edu.gr/aggregator/lo/photodentro-aggregatedcontent-8526-7189	Δημοτικό<br>Γυμνάσιο	Εικόνα	Γεωγραφία-Γεωλογία, Φυσικό περιβάλλον, Βιόσφαιρα, Ιστορία, Μυθολογία, Δωδεκάθεο, Εικαστικά, Ζωγραφική,<br>Θεατρική αγωγή, Ιστορία Θεάτρου Θεατρολογία
**8521/4054**	Τρως υγιεινά;	http://photodentro.edu.gr/aggregator/lo/photodentro-lor-8521-4054	Γυμνάσιο	Παρουσίαση, Γλωσσάρι	Γαλλικά , Γλωσσικές επικοινωνιακές ικανότητες, Λεξιλόγιο<br>Γαλλικά, Γενικές ικανότητες/δεξιότητες Κοινωνικο-πολιτισμικές γνώσεις<br>Γαλλικά, Γλωσσικές επικοινωνιακές ικανότητες, Ορθογραφία
**8522/812**	Ο Φρούτομαν ξαναχτυπά	http://photodentro.edu.gr/aggregator/lo/photodentro-educationalvideo-8522-812	Προσχολική, Δημοτικό Γυμνάσιο	Βίντεο	Αγωγή Υγείας
**8521/10751**	Βρες τις αναλογίες των ομάδων τροφίμων!	http://photodentro.edu.gr/aggregator/lo/photodentro-lor-8521-10751	Γυμνάσιο	Ασκήσεις πρακτικής και εξάσκησης	Φυσική αγωγή, Διατροφή

**8526/8281**	Η Ελιά ως πηγή ζωής, πολιτισμού και τέχνης	http://photodentro.edu.gr/aggregator/lo/photodentro-aggregatedcontent-8526-8281	Γυμνάσιο	Εκπαιδευτικό σενάριο, σχέδιο μαθήματος	Χωρίς αντιστοίχιση
**8526/5965**	Μαλλί της γριάς	http://photodentro.edu.gr/aggregator/lo/photodentro-aggregatedcontent-8526-5965	Δημοτικό Γυμνάσιο<br>ΕΠΑ.Λ	Εικόνα	Αγωγή Υγείας, Διατροφή, Διατροφικές συνήθειες,<br>Φυσική, Δυνάμεις και Κινήσεις, Γωνιακή ταχύτητα
**8526/6133**	Δέντρα με φρούτα στην Αίγυπτο	http://photodentro.edu.gr/aggregator/lo/photodentro-aggregatedcontent-8526-6133	Δημοτικό Γυμνάσιο	Εικόνα	Γεωγραφία – Γεωλογία, Φυσικό περιβάλλον, Πλανήτης Γη,<br>Αγωγή Υγείας, Διατροφή, Ομάδες τροφίμων,<br>Εικαστικά, Ζωγραφική
**8522/813**	Τρέφομαι Μεσογειακά	http://photodentro.edu.gr/aggregator/lo/photodentro-educationalvideo-8522-813	Προσχολική Δημοτικό Γυμνάσιο	Βίντεο	Αγωγή Υγείας
**8526/7892**	Βελτιώνοντας διατροφικές συνήθειες εφήβων	http://photodentro.edu.gr/aggregator/lo/photodentro-aggregatedcontent-8526-7892	Γυμνάσιο	Εκπαιδευτικό σενάριο - Σχέδιο μαθήματος	Αγωγή Υγείας, Διατροφή, Διατροφικές συνήθειες
**8531/312**	Κότινος	http://photodentro.edu.gr/aggregator/lo/photodentro-aggregatedcontent-8531-312	Γυμνάσιο	Εφαρμογή	Φυσική αγωγή
**8526/4479**	Θερισμός	http://photodentro.edu.gr/aggregator/lo/photodentro-aggregatedcontent-8526-4479	Προσχολική Δημοτικό<br>Γυμνάσιο	Εικόνα	Αγωγή Υγείας, Διατροφή, Ομάδες τροφίμων
**8522/22**	Υγιεινό μαγείρεμα	http://photodentro.edu.gr/aggregator/lo/photodentro-educationalvideo-8522-22	Γυμνάσιο<br>ΓΕΛ	Βίντεο	Αγωγή Υγείας

**8521/7459**	Η καθημερινή ζωή στην αρχαία Ελλάδα	http://photodentro.edu.gr/aggregator/lo/photodentro-lor-8521-7459	Γυμνάσιο<br><br>ΓΕΛ<br><br>ΕΠΑ.Λ	Ανοιχτή δραστηριότητα, Διερεύνηση, Εκπαιδευτικό παιχνίδι, Ιστοσελίδα	Αρχαία Ελληνική Γλώσσα και Γραμματεία, Άνθρωπος - ανθρώπινες σχέσεις και αξίες, Αθλητισμός, Διατροφή
**8521/10755**	Αξιολόγησε τις διατροφικές σου συνήθειες!	http://photodentro.edu.gr/aggregator/lo/photodentro-lor-8521-10755	Δημοτικό<br>Γυμνάσιο<br><br>ΓΕΛ	Βίντεο, Ασκήσεις πρακτικής και εξάσκησης	Φυσική αγωγή, Γνώσεις, Διατροφή, Φυσική αγωγή, Νοητικές δεξιότητες, Στοχοθεσία
**8521/11194**	Δείπνο στο Χρισσό (1805-1806)	http://photodentro.edu.gr/aggregator/lo/photodentro-lor-8521-11194	Δημοτικό<br>Γυμνάσιο<br><br>ΓΕΛ	Έργο τέχνης, Εξερεύνηση	Νεότερη και Σύγχρονη Ιστορία, Κοινωνία και καθημερινή ζωή
**8522/810**	Παραδοσιακές συνταγές Λεπτοκαρυάς Πιερίας	http://photodentro.edu.gr/aggregator/lo/photodentro-educationalvideo-8522-810	Δημοτικό<br>Γυμνάσιο<br><br>ΕΠΑ.Λ	Βίντεο	Αγωγή Υγείας
**8521/10750**	Η πίτα της διατροφής	http://photodentro.edu.gr/aggregator/lo/photodentro-lor-8521-10750	Γυμνάσιο	Ασκήσεις πρακτικής και εξάσκησης	Φυσική αγωγή, Γνώσεις, Διατροφή
**8526/7973**	Ευρωπαϊκή ποδηλατική διαδρομή EuroVelo 6:<br><br>Η αποστολή μας	http://photodentro.edu.gr/aggregator/lo/photodentro-aggregatedcontent-8526-7973	Γυμνάσιο	Εκπαιδευτικό σενάριο - Σχέδιο μαθήματος	Χωρίς αντιστοίχιση
**8526/7907**	Ζώντας και πεθαίνοντας στο Βυζάντιο	http://photodentro.edu.gr/aggregator/lo/photodentro-aggregatedcontent-8526-7907	Γυμνάσιο	Εκπαιδευτικό σενάριο - Σχέδιο μαθήματος	Βυζαντινή και Μεσαιωνική Ιστορία, Κοινωνική οργάνωση και καθημερινή ζωή

**8521/11213**	Διακρίνω πρωτογενείς και δευτερογενείς ιστορικές πηγές για τις συνθήκες διαβίωσης στην Κατοχή	http://photodentro.edu.gr/aggregator/lo/photodentro-lor-8521-11213	Δημοτικό Γυμνάσιο	Εξερεύνηση, Ασκήσεις πρακτικής και εξάσκησης, Παρουσίαση	Νεότερη και Σύγχρονη Ιστορία, Κοινωνία και καθημερινή ζωή
**8521/11206**	Ιστορικά θέματα και πηγές - Διάκριση θεματικών πεδίων κοινωνικής ιστορίας του 19ου αιώνα	http://photodentro.edu.gr/aggregator/lo/photodentro-lor-8521-11206	Γυμνάσιο<br>ΓΕΛ	Διερεύνηση	Ιστορία, Νεότερη και Σύγχρονη Ιστορία
**8522/184**	Φάσεις της βιομηχανίας τροφίμων	http://photodentro.edu.gr/aggregator/lo/photodentro-educationalvideo-8522-184	Γυμνάσιο<br>ΓΕΛ	Βίντεο	Σχεδιασμός και Τεχνολογία, Παραγωγή, Προϊόντα
**8521/11207**	Ιστορικά θέματα και πηγές - Διάκριση θεματικών πεδίων κοινωνικής ιστορίας του Μεσοπολέμου	http://photodentro.edu.gr/aggregator/lo/photodentro-lor-8521-11207	Γυμνάσιο<br>ΓΕΛ	Διερεύνηση	Νεότερη και Σύγχρονη Ιστορία, Πολιτικά και Ιδεολογικά Ζητήματα, Κοινωνία και καθημερινή ζωή
**8526/7962**	Κίνηση και στήριξη στον άνθρωπο	http://photodentro.edu.gr/aggregator/lo/photodentro-aggregatedcontent-8526-7962	Γυμνάσιο	Εκπαιδευτικό σενάριο - Σχέδιο μαθήματος	Βιολογία, Κίνηση – Στήριξη, Άνθρωπος - Μυοσκελετικό σύστημα
**8526/8218**	"MAKE YOUR VOICE HEARD"	http://photodentro.edu.gr/aggregator/lo/photodentro-aggregatedcontent-8526-8218	ΓΕΛ	Εκπαιδευτικό σενάριο - Σχέδιο μαθήματος	Αγγλικά, ΜΜΕ, Παραγωγή γραπτού λόγου
**8526/8216**	Η αναπαραγωγή στον άνθρωπο , Από τη	http://photodentro.edu.gr/aggregator/lo/photodentro-aggregatedcontent-8526-8216	Γυμνάσιο	Εκπαιδευτικό σενάριο - Σχέδιο μαθήματος	Βιολογία, Αναπαραγωγή, Άνθρωπος -

	γονιμοποίηση στη γέννηση με χρήση ιστοσελίδων και φύλλων εργασίας				Αναπαραγωγικό σύστημα
**8526/7995**	Αυτό το αλάτι της αλατιέρας εγώ το είδα στις αλυκές	http://photodentro.edu.gr/aggregator/lo/photodentro-aggregatedcontent-8526-7995	Γυμνάσιο	Εκπαιδευτικό σενάριο - Σχέδιο μαθήματος	Περιβαλλοντική Εκπαίδευση / Εκπαίδευση για την Αειφόρο Ανάπτυξη, Χερσαία Οικοσυστήματα & Οικοσυστήματα Εσωτερικών Υδάτων
**8526/3009**	Στρατιώτες στη σειρά για το συσσίτιο, Αλβανικό μέτωπο, 1940	http://photodentro.edu.gr/aggregator/lo/photodentro-aggregatedcontent-8526-3009	Δημοτικό, Γυμνάσιο<br><br>ΓΕΛ	Εικόνα	Νεότερη και Σύγχρονη Ιστορία, Πολεμικά γεγονότα
**8526/883**	Αλβανικό μέτωπο, ώρα φαγητού	http://photodentro.edu.gr/aggregator/lo/photodentro-aggregatedcontent-8526-883	Δημοτικό, Γυμνάσιο<br><br>ΓΕΛ<br><br>ΕΠΑ.Λ	Εικόνα	Νεότερη και Σύγχρονη Ιστορία, Πολεμικά γεγονότα, Εικαστικά, Οπτικοακουστικές Τέχνες
**8526/375**	Ο στρατιώτης Κοκκίνης την ώρα του φαγητού. Αλβανικό μέτωπο, 1940	http://photodentro.edu.gr/aggregator/lo/photodentro-aggregatedcontent-8526-375	Δημοτικό Γυμνάσιο	Εικόνα	Νεότερη και Σύγχρονη Ιστορία, Πολεμικά γεγονότα
**8521/3988**	Βρες την σπεσιαλιτέ/το τρόφιμο	http://photodentro.edu.gr/aggregator/lo/photodentro-lor-8521-3988	Δημοτικό Γυμνάσιο	Εκπαιδευτικό παιχνίδι	Γαλλικά, Γλωσσικές επικοινωνιακές ικανότητες, Ορθογραφία, δεξιότητες, Κοινωνικο-πολιτισμικές γνώσεις

**8526/491**	Συσσίτιο στρατιωτών. Αλβανικό μέτωπο 1940	http://photodentro.edu.gr/aggregator/lo/photodentro-aggregatedcontent-8526-491	Δημοτικό Γυμνάσιο	Εικόνα	Νεότερη και Σύγχρονη Ιστορία, Πολεμικά γεγονότα
**8521/4058**	Έλα να δοκιμάσεις τις σπεσιαλιτέ μου!	http://photodentro.edu.gr/aggregator/lo/photodentro-lor-8521-4058	Γυμνάσιο<br><br>ΓΕΛ	Εικόνα, Κείμενο, Βίντεο, Παρουσίαση	Γαλλικά, Γλωσσικές επικοινωνιακές ικανότητες – εκφράσεις, Δομή λόγου/κειμένου<br><br>Λεξιλόγιο, Γενικές δεξιότητες, Κοινωνικο-πολιτισμικές γνώσεις
**8522/535**	Περιγραφή της βουλιμίας	http://photodentro.edu.gr/aggregator/lo/photodentro-educationalvideo-8522-535	Γυμνάσιο<br><br>ΓΕΛ	Βίντεο	Αγωγή Υγείας
**8526/7900**	Ομάδες τροφίμων	http://photodentro.edu.gr/aggregator/lo/photodentro-aggregatedcontent-8526-7900	Γυμνάσιο	Εκπαιδευτικό σενάριο, Σχέδιο μαθήματος	Οικιακή Οικονομία
**8526/1725**	Φράουλα, πίνακας του Hortala Philippe	http://photodentro.edu.gr/aggregator/lo/photodentro-aggregatedcontent-8526-1725	Γυμνάσιο<br><br>ΓΕΛ	Εικόνα,<br><br>Πηγή - αναφορά	Εικαστικά, Ζωγραφική, Αγωγή Υγείας, Διατροφή, Καταναλωτικά πρότυπα
**8521/11091**	Υδατικό αποτύπωμα - Πόσο νερό «έφαγες» σήμερα;	http://photodentro.edu.gr/aggregator/lo/photodentro-lor-8521-11091	Δημοτικό, Γυμνάσιο<br><br>ΓΕΛ<br><br>ΕΠΑ.Λ	ασκήσεις πρακτικής και εξάσκησης, εκπαιδευτικό παιχνίδι, παρουσίαση	Περιβαλλοντική Εκπαίδευση, Αειφόρος Ανάπτυξη, Νερό, Παραγωγή & Κατανάλωση
**8526/5762**	Συσσίτιο σε ίδρυμα για παιδιά	http://photodentro.edu.gr/aggregator/lo/photodentro-aggregatedcontent-8526-5762	Δημοτικό Γυμνάσιο	Εικόνα	Αγωγή Υγείας, Διατροφή, Γεύματα

**8526/2910**	Παναγιάριο, Φιλοξενία του Αβραάμ	http://photodentro.edu.gr/aggregator/lo/photodentro-aggregatedcontent-8526-2910	Δημοτικό Γυμνάσιο	Εικόνα	Θρησκευτικά, Εκκλησιαστική Τέχνη, Ζωγραφική
**8526/5046**	Πανδοχείο στην Κόρινθο	http://photodentro.edu.gr/aggregator/lo/photodentro-aggregatedcontent-8526-5046	Δημοτικό Γυμνάσιο<br><br>ΕΠΑ.Λ	Εικόνα	Νεότερη και Σύγχρονη Ιστορία (19ος-20ός αι.), Κοινωνία και καθημερινή ζωή, Εικαστικά, Ζωγραφική<br><br>Πολεμικά γεγονότα,<br><br>Διαπολιτισμική Αγωγή
**8526/539**	Ορφανό κορίτσι	http://photodentro.edu.gr/aggregator/lo/photodentro-aggregatedcontent-8526-539	Δημοτικό Γυμνάσιο<br><br>ΓΕΛ	Εικόνα	Νεότερη και Σύγχρονη Ιστορία (19ος-20ός αι.), Κοινωνία - καθημερινή ζωή
**8522/710**	Του Δάσους Μυρωδιές, του Νερού Κελάρυσμα	http://photodentro.edu.gr/aggregator/lo/photodentro-educationalvideo-8522-710	Δημοτικό<br><br>Γυμνάσιο<br><br>ΓΕΛ<br><br>ΕΠΑ.Λ	Βίντεο	Σχεδιασμός και Τεχνολογία<br><br>Περιβαλλοντική Εκπαίδευση - Αειφόρο Ανάπτυξη, Παραγωγή & Κατανάλωση<br><br>Χερσαία Οικοσυστήματα & Εσωτερικών Υδάτων
**8526/5901**	Διανομή συσσιτίου - Ήπειρος 1912-1913	http://photodentro.edu.gr/aggregator/lo/photodentro-aggregatedcontent-8526-5901	Δημοτικό<br><br>Γυμνάσιο<br><br>ΓΕΛ<br><br>ΕΠΑ.Λ	Εικόνα	Νέα Ελληνική Γλώσσα, Κειμενικά είδη, Κείμενα σχολιαστικά, ΤΠΕ, Διαδίκτυο, Θεατρική αγωγή, Δραστηριότητες

					Αγωγή Υγείας, Διατροφή, Διατροφικές συνήθειες
**8526/4469**	Ανεμόμυλος στη Σκύρο	http://photodentro.edu.gr/aggregator/lo/photodentro-aggregatedcontent-8526-4469	Δημοτικό Γυμνάσιο	Εικόνα	Γεωγραφία – Γεωλογία, Ανθρωπογενές περιβάλλον, Ανάπτυξη
**8526/4090**	Κάτοψη αρχαίου ελληνικού σπιτιού	http://photodentro.edu.gr/aggregator/lo/photodentro-aggregatedcontent-8526-4090	Γυμνάσιο<br>ΓΕΛ	Εικόνα	Ιστορία, Κοινωνική οργάνωση, Καθημερινή ζωή<br>Πολιτικές και κοινωνικές επιστήμες
**8526/2994**	Ο Πολλαπλασιασμός των Άρτων	http://photodentro.edu.gr/aggregator/lo/photodentro-aggregatedcontent-8526-2994	Δημοτικό Γυμνάσιο	Εικόνα	Θρησκευτικά, Καινή Διαθήκη, Εικαστικά, Ζωγραφική
**8526/6487**	Υπαίθριο εστιατόριο στην Κωνσταντινούπολη	http://photodentro.edu.gr/aggregator/lo/photodentro-aggregatedcontent-8526-6487	Δημοτικό Γυμνάσιο<br>ΓΕΛ<br>ΕΠΑ.Λ	Εικόνα	Νέα Ελληνική Γλώσσα, Ανθρώπινες σχέσεις, Νεότερη και Σύγχρονη Ιστορία, Κοινωνία και καθημερινή ζωή, Αγωγή Υγείας, Διατροφή
**8526/5266**	Σχέδιο του O'Netty με θέμα εστιατόριο στο δρόμο της Κωνσταντινούπολης	http://photodentro.edu.gr/aggregator/lo/photodentro-aggregatedcontent-8526-5266	Δημοτικό Γυμνάσιο<br>ΓΕΛ<br>ΕΠΑ.Λ	Εικόνα,<br>Έργο τέχνης	Εικαστικά, Σχέδιο-χρώμα<br>Νεότερη και Σύγχρονη Ιστορία, Κοινωνία και καθημερινή ζωή
**8526/5055**	Μαθητές στο Στρούνι Ιωαννίνων	http://photodentro.edu.gr/aggregator/lo/photodentro-aggregatedcontent-8526-5055	Δημοτικό Γυμνάσιο	Εικόνα	Νεότερη και Σύγχρονη Ιστορία, Τέχνες, Γράμματα και Πολιτισμός

					Γεωγραφία – Γεωλογία, Ανθρωπογενές περιβάλλον, Κοινωνική ζωή και Πολιτισμός<br><br>Νέα Ελληνική Γλώσσα, Παιδεία - Σχολική ζωή
**8526/8322**	Nutrition facts & Food Labels	http://photodentro.edu.gr/aggregator/lo/photodentro-aggregatedcontent-8526-8322	ΓΕΛ	Εκπαιδευτικό σενάριο - Σχέδιο μαθήματος	Αγγλικά, Διατροφή και αγορά, Κατανόηση γραπτού λόγου
**8526/5048**	Τουρκάλες της Μικράς Ασίας ζυμώνουν ψωμί	http://photodentro.edu.gr/aggregator/lo/photodentro-aggregatedcontent-8526-5048	Δημοτικό Γυμνάσιο<br><br>ΓΕΛ	Εικόνα	Νεότερη και Σύγχρονη Ιστορία, Κοινωνία και καθημερινή ζωή<br><br>Εικαστικά, Ζωγραφική,<br><br>Αγωγή Υγείας, Διατροφή, Διατροφικές συνήθειες,<br><br>Νέα Ελληνική Γλώσσα,<br><br>Πολιτικά και Ιδεολογικά Ζητήματα
**8526/7616**	ΧΑΛΚΟΥΡΓΕΙΟ, ΜΥΤΙΛΗΝΗ	http://photodentro.edu.gr/aggregator/lo/photodentro-aggregatedcontent-8526-7616	Δημοτικό<br><br>Γυμνάσιο<br><br>ΓΕΛ	Εικόνα	Νεότερη και Σύγχρονη Ιστορία, Τέχνες, Γράμματα και Πολιτισμός
**8526/4420**	Ζωοπανήγυρις στη Μακεδονία	http://photodentro.edu.gr/aggregator/lo/photodentro-aggregatedcontent-8526-4420	Δημοτικό Γυμνάσιο<br><br>ΓΕΛ	Εικόνα	Εικαστικές Τέχνες και Τεχνολογία<br><br>Μαθηματικά, Αριθμητική,

					Νεότερη και Σύγχρονη Ιστορία, Οικονομία, Κοινωνία και καθημερινή ζωή
**8521/11077**	Ψώνια στο οπωροπωλείο	http://photodentro.edu.gr/aggregator/lo/photodentro-lor-8521-11077	Δημοτικό Γυμνάσιο	Μικροπείραμα εκπαιδευτικό παιχνίδι	Μαθηματικά, Αριθμητική, Λόγοι – Αναλογίες, Μέτρηση βάρους
**8526/5918**	Διανομή συσσιτίου την περίοδο της Κατοχής	http://photodentro.edu.gr/aggregator/lo/photodentro-aggregatedcontent-8526-5918	Δημοτικό Γυμνάσιο	Εικόνα	Νεότερη και Σύγχρονη Ιστορία, Πολεμικά γεγονότα, Οικονομία, Κοινωνία και καθημερινή ζωή
**8526/8465**	Εισαγωγή στα μακρομόρια	http://photodentro.edu.gr/aggregator/lo/photodentro-aggregatedcontent-8526-8465	Γυμνάσιο	Εκπαιδευτικό σενάριο - Σχέδιο μαθήματος	Βιολογία, Βιοχημεία, Βιομόρια
**8526/5956**	Κέντρο γάλακτος Διεθνούς Ερυθρού Σταυρού στην Κατοχή	http://photodentro.edu.gr/aggregator/lo/photodentro-aggregatedcontent-8526-5956	Δημοτικό Γυμνάσιο	Εικόνα	Νεότερη και Σύγχρονη Ιστορία, Πολεμικά γεγονότα
**8526/6468**	Διανομή ψωμιού στην Αθήνα την περίοδο της Κατοχής,	http://photodentro.edu.gr/aggregator/lo/photodentro-aggregatedcontent-8526-6468	Δημοτικό Γυμνάσιο	Εικόνα	Νεότερη και Σύγχρονη Ιστορία, Πολεμικά γεγονότα, Οικονομία
**8526/4354**	Άποψη της Ζακύνθου με το ενετικό κάστρο	http://photodentro.edu.gr/aggregator/lo/photodentro-aggregatedcontent-8526-4354	Προσχολική Δημοτικό Γυμνάσιο ΓΕΛ	Εικόνα, Παρουσίαση	Ιστορία, Νεότεροι χρόνοι, Οικονομία, Κοινωνική και πολιτική οργάνωση

**8521/4866**	Τα βασικά συστατικά των τροφίμων	http://photodentro.edu.gr/aggregator/lo/photodentro-lor-8521-4866	Δημοτικό Γυμνάσιο	Παρουσίαση, Γλωσσάρι	Βιολογία, Άνθρωπος και Υγεία, Διατροφή
**8522/1224**	Παιδική παχυσαρκία	http://photodentro.edu.gr/aggregator/lo/photodentro-educationalvideo-8522-1224	Γυμνάσιο<br>ΓΕΛ<br>ΕΠΑ.Λ<br>Ειδική Αγωγή	Βίντεο	Αγωγή Υγείας, Διατροφή, Παχυσαρκία
**8522/1228**	Βουλιμία	http://photodentro.edu.gr/aggregator/lo/photodentro-educationalvideo-8522-1228	Γυμνάσιο<br>ΓΕΛ<br>ΕΠΑ.Λ<br>Ειδική Αγωγή	Βίντεο	Αγωγή Υγείας, Διατροφή, Διατροφικές συνήθειες
**8522/1221**	Ανορεξία	http://photodentro.edu.gr/aggregator/lo/photodentro-educationalvideo-8522-1221	Γυμνάσιο<br>ΓΕΛ<br>ΕΠΑ.Λ	Βίντεο	Αγωγή Υγείας, Διατροφή, Διατροφικές συνήθειες

For each learning object, the following information is provided: title, URL (resource page), unique identifier code, educational level, type of resource, and thematic classification.
